# Genotype and phenotype of balb/c mouse strain expressing h-2k^b^-tsa58- sv40 immortalizing oncogene

**DOI:** 10.1186/1753-6561-7-S2-P3

**Published:** 2013-04-04

**Authors:** Aline C Custódio, Fabiane CF Brito, Mara S Junqueira, Roger Chammas, José E Belizário

**Affiliations:** 1 Department of Pharmacology, Institute of Biomedical Sciences, University of São Paulo, Brazil; 2 Department of Radiology and Oncology, Medical School of University of São Paulo, Brazil

## Introduction

The Simian Virus 40 (SV40) large T antigen is multifunctional protein with DNA helicase, RNA helicase and ATPse activities which contribute to multistep tumorogenesis in rodents and humans. The Immortomouse mouse strain expresses a mutated large T antigen tsA58 oncogene under the control of the interferon inducible murine H-2K^b^ promoter on chromosome 16. Our aim was to establish a BALB/c strain of H-2K^b^-tsA58 immortomice that could be utilized to investigate specific pathological and physiological patterns associated SV-40 oncogenicity and generation of conditionally immortal cells lines.

## Methods and results

We have crossed H2K^b^-SV40-tsA58 CBA/CaxC57BL/10 hybrid immortomice with BALB/c mice to obtain a transgenic colony with unique BALB/c background. We have used two pre-validated PCR genotype assays that can distinguish between wild-type, hemizygous, and homozygous animals (4-6 months old). Enlargement of thymus is a phenotypic abnormality of immortomouse. We have characterized macroscopically and by immunohistochemistry in the F1-3 offspring hemizygous females with thymic hyperplasia (2:5 ratio). Studies are underway to typing T cell (CD4/8) populations in the thymuses. Moreover, we observed four small males displaying abnormality after birth.

## Conclusion

This transgenic mouse strain will help to isolate immortalizing cell lines growing under the permissive 33°C temperature for the studies of the SV40 large T antigen transformation.

## Financial support

CNPq, ICGEB.

